# Automated Quantitative Analysis of CT Perfusion to Classify Vascular Phenotypes of Pancreatic Ductal Adenocarcinoma

**DOI:** 10.3390/cancers16030577

**Published:** 2024-01-30

**Authors:** Tom Perik, Natália Alves, John J. Hermans, Henkjan Huisman

**Affiliations:** Department of Medical Imaging, Radboud University Medical Center, 6525 GA Nijmegen, The Netherlandsjohn.hermans@radboudumc.nl (J.J.H.); henkjan.huisman@radboudumc.nl (H.H.)

**Keywords:** perfusion imaging, biomarker, pancreatic neoplasms

## Abstract

**Simple Summary:**

This study introduces a novel semi-automated artificial intelligence (AI) biomarker for CT perfusion (CTP) analysis with the goal of identifying vascular phenotypes of pancreatic ductal adenocarcinoma (PDAC) and assessing their effects on overall survival (OS). A deep learning approach combined with a CTP curve model was utilized. The biomarker successfully differentiated tumor vascular characteristics among 86 analyzed PDAC patients. Notably, tumors identified as isovascular by the AI biomarker showed distinct enhancement metrics. These vascular phenotypic differences were significantly associated with variations in OS. Thus, AI-enhanced CTP analysis seems a promising potential clinical tool for predicting prognoses in PDAC patients.

**Abstract:**

CT perfusion (CTP) analysis is difficult to implement in clinical practice. Therefore, we investigated a novel semi-automated CTP AI biomarker and applied it to identify vascular phenotypes of pancreatic ductal adenocarcinoma (PDAC) and evaluate their association with overall survival (OS). Methods: From January 2018 to November 2022, 107 PDAC patients were prospectively included, who needed to undergo CTP and a diagnostic contrast-enhanced CT (CECT). We developed a semi-automated CTP AI biomarker, through a process that involved deformable image registration, a deep learning segmentation model of tumor and pancreas parenchyma volume, and a trilinear non-parametric CTP curve model to extract the enhancement slope and peak enhancement in segmented tumors and pancreas. The biomarker was validated in terms of its use to predict vascular phenotypes and their association with OS. A receiver operating characteristic (ROC) analysis with five-fold cross-validation was performed. OS was assessed with Kaplan–Meier curves. Differences between phenotypes were tested using the Mann–Whitney U test. Results: The final analysis included 92 patients, in whom 20 tumors (21%) were visually isovascular. The AI biomarker effectively discriminated tumor types, and isovascular tumors showed higher enhancement slopes (2.9 Hounsfield unit HU/s vs. 2.0 HU/s, *p* < 0.001) and peak enhancement (70 HU vs. 47 HU, *p* < 0.001); the AUC was 0.86. The AI biomarker’s vascular phenotype significantly differed in OS (*p* < 0.01). Conclusions: The AI biomarker offers a promising tool for robust CTP analysis. In PDAC, it can distinguish vascular phenotypes with significant OS prognostication.

## 1. Introduction

Dynamic CT perfusion (CTP) is a functional imaging technique that enables the quantification of tissue vascularization [1]. CTP is based on temporal changes in tissue attenuation after the injection of a contrast medium. Mathematical models can be used to calculate the quantitative features of tissue. Early applications of CTP were focused on brain imaging, but in recent years, multiple studies have used CTP for oncologic imaging [2]. Oncological applications involve lesion characterization, prognostication, and treatment response evaluation [1,3,4,5]. Previous studies have shown that CTP can also help in the detection and treatment assessment of pancreatic ductal adenocarcinoma (PDAC) [6,7,8,9].

Pancreatic ductal adenocarcinoma (PDAC) is the third leading cause of cancer-related death in the USA. Its prognosis is poor, with a five-year survival rate of 12% [10,11]. The treatment of PDAC poses clinical challenges, especially in tailoring optimal treatment strategies. Prognostication could help in selecting subtypes with different treatment options. Although the majority of tumors are hypoattenuating, a substantial subgroup, ranging from 10 to 20%, is isoattenuating [12,13,14]. Earlier studies demonstrated that isoattenuating tumors showed significantly longer survival after resection [12,15]. However, the detection of isoattenuating tumors is difficult because there is no clear demarcation between tumor tissue and pancreatic parenchyma. Therefore, secondary signs are needed to detect these tumors [13,16]. The quantification of perfusion using CTP can enable the improved classification of these vascular subtypes. This information can be valuable in shared decision-making and associated quality of life.

The clinical implementation of CTP for oncology is still limited because of a lack of consistent kinetic models, and the analysis of CTP is time-consuming. Perfusion parameters rely heavily on the specific kinetic models applied, which restricts their interchangeability and interpretation, thus impeding their use in clinical decision-making [8,9,17,18]. Previous perfusion studies used manual annotations to create a region of interest (ROI), which was time-consuming and user-dependent, hampering its practical integration into clinical routine [4,6,19,20].

To address these issues, we developed a novel method for the automatic quantification of tumor perfusion with CTP based on a patented pharmacokinetic dynamic contrast-enhanced MRI (DCE-MRI) analysis of prostate cancer [21]. We hypothesized that a kinetic-model-independent method would allow for a consistent time–intensity curve (TIC) analysis. Furthermore, we used a deep learning segmentation model to automate ROI selection [22]. This enabled user-independent and rapid ROI selection of the entire tumor, to calculate perfusion features in all tumors.

This study was aimed at evaluating our automated CTP analysis for identifying vascular phenotypes of PDAC and correlating vascular phenotypes with overall survival in patients with PDAC.

## 2. Materials and Methods

### 2.1. Patients

Institutional review board approval for this single-center study was obtained, and all patients gave written informed consent. All inclusions and scans were performed at the Radboud University Medical Center. Between January 2018 and September 2022, patients with a clinical suspicion of pancreatic cancer, as determined by the multidisciplinary tumor board, were prospectively included. The exclusion criteria were aged below 18 years, previous treatment for pancreatic cancer, concomitant malignancies, contraindications of CECT (i.e., untreatable contrast allergy and severe renal function impairment), and insufficient command of the Dutch language. Only patients with histopathological proof of PDAC, obtained via fine needle aspiration/biopsy (FNA/FNB) or tumor resection, were included in the final analysis. Furthermore, image quality needed to be sufficient for analyzing the time–intensity curve. If the entire tumor was not captured in scan volume, or the quality of motion correction after registration was insufficient, the patient was excluded. Patients with a loss of follow-up within 6 months were excluded from the final analysis. Clinical data were gathered from electronic patient records. This study is registered in the US National Library of Medicine registry (https://clinicaltrials.gov/, accessed on 19 December 2023) under identifier: NCT05669287.

### 2.2. Image Acquisition

All CT exams were performed on a 160 mm wide detector 320-row scanner ( Canon Aquilion ONE Genesis Edition, Canon Medical Systems Corporation, Ōtawara, Japan). The amount of contrast agent (400 mg/iomeprol, lomeron, Bracco) was body-weight-based (1.3 mg I/kg) and intravenously administered at the fixed-duration injection time of 5 s. A test bolus of 15 mL was used to determine the time of contrast arrival in the aorta at the level of the pancreas. A 5 mL saline bolus chase was injected directly after the contrast agent injection.

**Figure 1:**
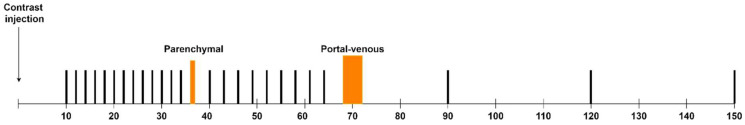
The interleaved CT perfusion protocol. All black bars represent dynamic perfusion acquisition. The orange bars show the interleaved diagnostic protocol in the non-contrast-enhanced, parenchymal, and portal-venous phases.

Based on the time bolus, the perfusion series was started 2 s before the arrival time of the measured contrast in the aorta, with 13 acquisitions at 2 s intervals, followed by a diagnostic volume scan in the parenchymal phase at around 35 s and 9 dynamic acquisitions at 3 s intervals. At 70 s, a portal-venous diagnostic helical scan of the thorax and abdomen was performed, followed by three dynamic acquisitions at 90, 120, and 150 s. Only the upper abdomen was included in the perfusion scans and in the diagnostic scan in the parenchymal phase. The complete scan protocol is shown in Figure 1. Combining perfusion CT with diagnostic CT, the interleaved scanning protocol allowed for obtaining both quantitative information and staging information with a single intravenous contrast agent injection. This “one-stop shop” protocol acquired the maximum diagnostic information in a patient-friendly and time- and cost-efficient manner, which facilitates the use of CTP in routine clinical practice [23,24].

### 2.3. CTP AI Biomarker

**Figure 2:**
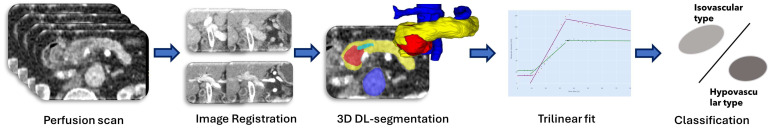
Modules of the CTP AI biomarker, starting on the left with the perfusion scan as the input. The first module step was image registration, followed by 3D deep learning segmentation. This segmentation was used to create the TIC, which was fitted via trilinear fit to calculate the perfusion features. These features were used in a classification algorithm to discriminate vascular phenotypes.

The novel biomarker comprised a pipeline of modules to transform a CTP image into a lesion phenotype prediction, as shown in Figure 2. The first module in the pipeline was a non-rigid image registration using Body Registration software version 10.0.0 (Canon Medical Systems Corporation).

The second module was a fully automated deep learning model (nnUnet framework) previously developed in-house to create a 3D segmentation of tumor and pancreas parenchyma [22]. This model was trained with portal-venous CT scans of patients with histopathologically proven PDAC of the pancreatic head. To train manual annotations of the tumor, the following were used: pancreas parenchyma, portomesenteric and splenic veins, abdominal aorta, celiac trunk, hepatic, splenic, and superior mesenteric arteries, pancreatic duct, and common bile duct.

The deep learning model was originally trained on CT scans of the portal-venous phase. For CTP analysis the last acquisition before the portal-venous phase was then applied as the input for this model. A fixed-size volume of interest was used to extract the region around the pancreas, serving as input for the segmentation model. The output wasa 3D segmentation of the tumor and multiple surrounding anatomical structures. The 3D segmentation of this timepoint was overlayed on all other timepoints in the perfusion sequence to enable both tumor and pancreas parenchyma to create a TIC as the input for the curve fit. All automated segmentations were checked by an experienced radiologist specialized in pancreatic imaging (J.H.). In the case of isoattenuating tumors, MRI was used to validate the tumor segmentation. The tumor segmentation was adjusted when the tumor location was incorrectly classified by the model. Vessels, endoprosthesis, and a self-expandable metal stent (SEMS) running through the tumor segmentation were not included in the segmentation to reduce artifacts in the TIC.

The third module was a newly developed automated trilinear curve fit based on an in-house-developed and patented pharmacokinetic analysis package for DCE-MRI in prostate cancer [21]. The trilinear model was focused on the first, upslope part of the curve to avoid recirculation effects. The curve fit comprised two fitting steps: First, a higher-order spline fit was used to robustly and automatically fit the complete time–intensity curve. This spline fit was used to identify the time zone of the first upslope by determining the maximum derivative. Second, the trilinear fit upslope phase was centered around this maximum derivative by selecting five timepoints surrounding the maximum derivative. The timepoints preceding and following the upslope phase were assigned to the non-enhancement phase and the washout phase, respectively. This strategy allowed us to generate a curve fit that separated three phases: the non-enhancement phase, the upslope phase, and the washout phase. The intersection of the enhancement slope and the static line was the start of enhancement. The peak enhancement was calculated as the intersection of the washout phase with the enhancement phase. Using this method, we calculated three main perfusion features: slope, peak enhancement, and the difference in enhancement between the tumor and pancreatic parenchyma. The curve fit was repeated for the tumor and pancreas ROIs.

The final module comprised a machine learning module that was trained to discriminate two vascular phenotypes. To discriminate computational vascular phenotypes, a logistic regression model was trained using three perfusion features: tumor slope (HU/s), tumor peak enhancement (HU), and the difference between peak enhancement of tumor and peak enhancement of pancreas parenchyma (ΔHU). The visual phenotype classification was used as a reference. The logistic regression model was trained using five-fold cross-validation. To assess the model’s performance, receiver operating characteristic (ROC) curves were produced for each fold. In addition, area under the curve (AUC) scores were used to measure the model’s performance.

### 2.4. Visual Annotation

**Figure 3:**
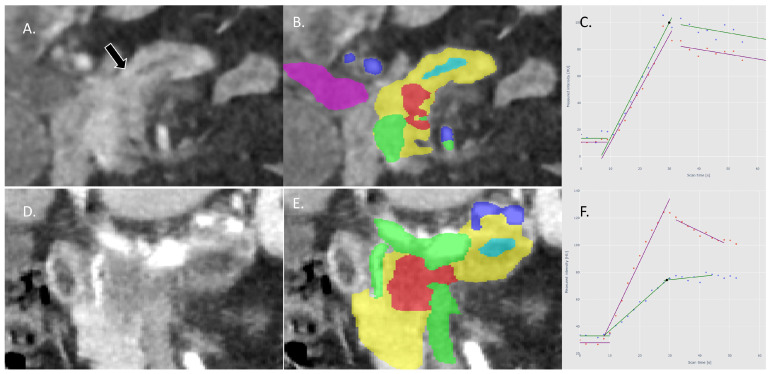
(**A**): Example of CTP image of a visual isovascular tumor in the coronal plane: no pancreatic lesion is visible, only signs of obstruction of the pancreatic duct (arrow). (**B**): Tumor (red) as segmented with the deep learning model; other segmentations are pancreas parenchyma (yellow), pancreatic duct (light blue), arteries (dark blue), portomesenteric and splenic veins (green), and the common bile duct (purple) (**C**): The TICs of the tumor (green) and pancreas (purple) are almost identical, showing similar perfusion in both tumor and pancreas parenchyma. (**D**,**E**): Example of a visual hypovascular tumor in the coronal plane with segmentations. (**F**): The TICs of the tumor (green) and pancreas (purple) with a significantly lower perfusion of the tumor compared with the pancreas parenchyma.

Visually isoattenuating or isovascular pancreatic adenocarcinoma was defined based on the following criteria: an obstructed pancreatic duct and/or common bile duct without a visible pancreatic lesion of increased or decreased attenuation, compared with pancreatic parenchyma, in both the arterial and portal-venous phases; the difference in attenuation between the pancreatic tumor and the adjacent upstream or downstream pancreas parenchyma was less than 10 HU [25]. Figure 3 shows examples of both visual isovascular and hypovascular tumors. All scans were annotated by an experienced radiologist specialized in pancreatic imaging (J.H.).

### 2.5. Statistical Analysis

Descriptive statistics were used to summarize the clinical characteristics of the study population. Continuous variables were expressed as mean ± standard deviation or median (interquartile range), depending on their distribution, which was assessed using the Shapiro–Wilk test. Categorical variables were expressed as frequencies and percentages.

Phenotype prediction was evaluated by assessing the differences in perfusion parameters. ROC analysis was used to assess the ability to predict the correct phenotype. A Mann–Whitney U test was used to determine the significance of differences between perfusion features. The chi-square test was used to assess clinical categorical features. Survival analysis was performed with a log-rank test using the lifelines package (version 0.28.0) in Python. A *p*-value less than 0.05 was considered statistically significant.

## 3. Results

### 3.1. Patient Cohort

**Figure 4:**
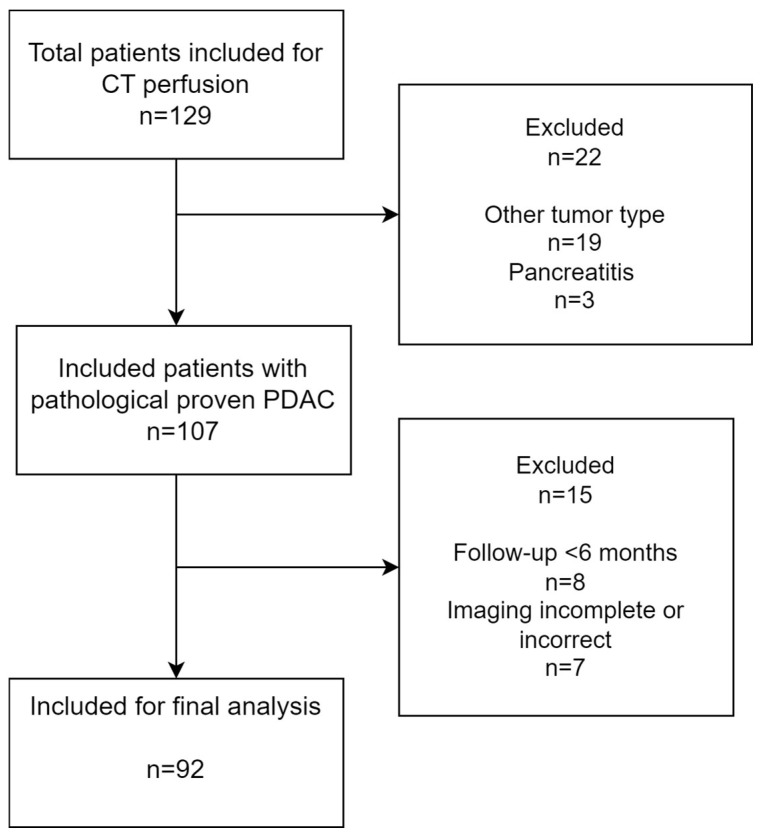
Inclusion flowchart of this study.

A total of 129 patients with clinical suspicion of pancreatic cancer were included. Of this group, 105 patients had histopathologically proven PDAC; 8 patients were excluded because of incomplete imaging (*n* = 7) and loss of follow-up within 1 year (*n* = 2). Therefore, for the final analysis, we included 92 patients with pathologically proven PDAC (mean age 60, 56% men), as shown in Figure 4. The median overall survival was 11.3 months, and the median follow-up period was 11.4 months.

Fully automated segmentation was possible for 76/92 (83%) of tumors; 16 tumors were not completely identified using the deep learning model. Of the unsuccessful segmentations, 9/16 tumors were tail tumors, and the other tumors were isovascular tumors in the pancreatic head. These segmentations were manually adjusted for inclusion in the analysis 

The clinical characteristics of these patients are summarized in Table 1.

### 3.2. Visual Phenotype

**Figure 5:**
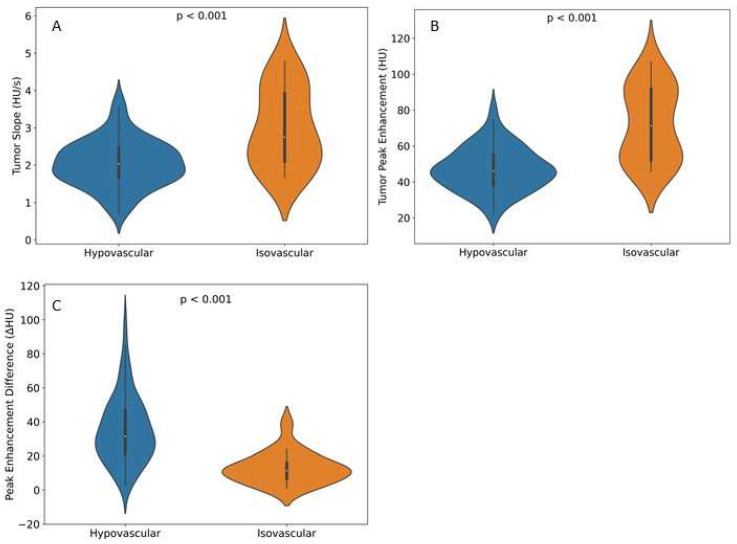
Violin plots showing perfusion features in both visual hypo- and isovascular tumors on the portal-venous phase of CECT. (**A**) Tumor slope measured in HU/s. (**B**) Tumor peak enhancement in HU for both tumor slope and tumor peak enhancement; visual isovascular tumors showed higher perfusion values compared with hypovascular tumors, with a significant difference in both features (*p* < 0.001). (**C**) Peak enhancement difference between tumor and non-tumorous pancreas parenchyma. Isovascular tumors showed a lower enhancement difference compared with hypovascular tumors, with a significant difference(*p* < 0.001).

Automated CTP features showed significant differences between visual phenotypes (Table 2). The mean enhancement slope was steeper in the isovascular tumors (2.9 ± 1.1 HU/s) compared with the hypovascular tumors (2.0 ± 0.6 HU/s) (*p* < 0.001). Additionally, the mean peak enhancement was found to be significantly higher in the isovascular tumors (69.9 ± 22.1 HU) compared with the hypovascular tumors (46.8 ± 12.2 HU) (*p* < 0.001). The difference between the peak enhancement of the tumor compared with the peak enhancement of the pancreas was also found to be lower in the isovascular tumors (12.0 ± 9.1 HU) compared with the hypovascular tumors (35.3 ± 19.4 HU), (*p* < 0.001), (Figure 5).

### 3.3. Computational Phenotype and Survival Prediction

**Figure 6:**
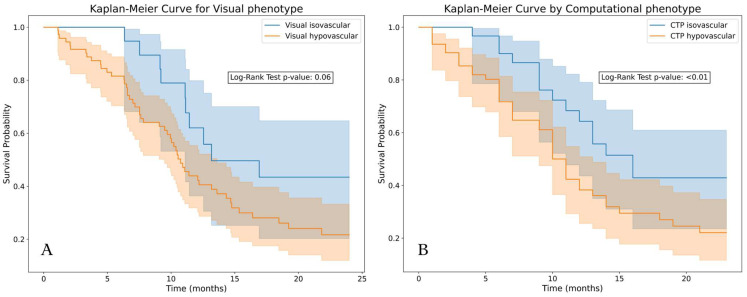
Kaplan–Meier plots of the tumors stratified by vascular type. (**A**): Based on visual classification, isovascular tumors showed longer survival (*p* = 0.06). (**B**): Based on a logistic regression trained on perfusion features (upslope tumor, peak enhancement tumor, and difference in peak enhancement), predicted isovascular tumors showed significantly longer survival (*p* < 0.01) compared with the predicted hypovascular tumors.

We found a significant difference in overall survival between the visual isovascular and hypovascular tumors and longer survival of patients with visual isovascular tumors (*p* = 0.06). The prediction of vascular phenotype had a mean AUC of 0.8, indicating that the computational phenotype could reproduce the visual phenotype predictions. Using the computational phenotype, we found a significantly longer survival of patients with tumors predicted as isovascular (*p* < 0.001) (Figure 6).

## 4. Discussion

The results showed that our novel AI biomarker for CT perfusion automatically identified vascular phenotypes in PDAC. The perfusion features of tumor slope and tumor peak enhancement were significantly higher in the visual isovascular tumor type compared with hypovascular tumors. The influence of tumor slope, which cannot be measured on conventional CT, was an additional novel CTP finding. The difference in the peak enhancement of tumor and pancreas was lower in the visual isovascular tumors, as expected. Furthermore, the results showed significant differences in OS for both the visual phenotype and the predicted phenotype of our AI biomarker, with prolonged survival of isovascular tumors. This finding is in line with an earlier study that showed improved survival of isovascular tumors classified on conventional CT and MRI [15].

Earlier studies demonstrated that CT perfusion can be used to determine histopathologic subtypes of PDAC or evaluate chemotherapy responses [6,26,27]. However, none of these studies investigated vascular subtypes or overall survival. Our study contributes new insights by demonstrating that the vascular subtypes defined by CTP can help with prognostication. Moreover, all prior CTP investigations utilized perfusion software that required the manual placement of ROIs [6,19,27,28]. Our automated method makes it possible to quantify perfusion of the entire tumor without the need for manual segmentation.

This study is the first to conduct an automated and kinetic-model-independent analysis of CTP in patients with PDAC. This approach could support the use of CTP in routine clinical settings, not only for PDAC but also for other tumors. The clinical evidence for the use of CTP is still limited, which could be explained by the difficulty in implementing the technique in a routine clinical setting.

Our AI biomarker depended on the CT acquisition protocol and multiple modules in the pipeline, all of which influenced the final results. The first module was the non-rigid registration model. Despite the non-rigid registration, because some residual movement was still present in the scans, a voxel-by-voxel analysis of the TIC was not possible. The automated segmentation was not correct in all cases, and therefore needed to be checked and adjusted manually if needed. Because the algorithm was trained on PDAC tumors in the pancreatic head, the tail tumors in particular required manual adjustment [22]. We used a single CTP timepoint to create the segmentation. In the case of poor registration, the tumor volume can move slightly over time, decreasing the stability of the TIC. Despite the occurrence of these problems in the first modules, the curve fit produced accurate results for tumor volume and pancreas parenchyma.

Multiple studies have shown that various commercial perfusion software generates significantly different perfusion features [8,29,30]. A reason could be the different boundary conditions of these kinetic models, which do not always reflect tumor tissue physiology. The advantage of our AI biomarker is that it does not require complex kinetic models, and it performs direct calculations on the TIC.

Although the results are promising, this study has the following limitations. First, although this research is the largest prospective study of CTP in PDAC to date, the study population was heterogenous, including different tumor stages and treatment strategies, which strongly influenced the survival data. A larger and more homogeneous sample size is required to validate our findings and correlate them with patient outcomes. Second, the current method is not yet fully automated because the 3D segmentations must be checked and adjusted in some cases. In particular, improvement in the AI-based segmentation of distal pancreas tumors could overcome the need for manual control.

Future research should explore the correlation between biomarker findings and pathological subtypes, which could lead to new insights into vascular tumor types. Another research direction could be the potential use of CT perfusion in the evaluation of responses to chemotherapy or, more specifically, evaluations of anti-angiogenic therapy.

## 5. Conclusions

Available CTP kinetic perfusion models are user-dependent, show poor discrimination power, and are difficult to implement in clinical practice. Because our new method is automatic and discriminative, it can facilitate the clinical adoption of CTP for functional oncological imaging.

## Figures and Tables

**Table 1:** Baseline characteristics of the study cohort.

Variable		Total (*n* = 92 Patients)	Visual Hypovascular (*n* = 72)	Visual Isovascular (*n* = 20)	*p*-Value *
Age (year)		66 ± 9	65 ± 9	68 ± 8	0.29
Sex (*n* = males)		51 (55%)	41 (59%)	39 (47%)	0.40
Tumor size (mm)		36 ± 16	39 ± 16	28 ± 13	0.01
Tumor location	Head Body–tail	60 (65%)	46 (66%)	14 (63%)	0.85
		32 (35%)	26 (34%)	6 (37%)	
Median overall survival (days)		337	320	377	0.04

* Tested using the Mann–Whitney U test; a chi-square test was used to determine clinical categorical features.

**Table 2:** Mean CT perfusion features of both visual vascular types.

Parameter	Visual Hypovascular	Visual Isovascular	*p*-Value
Tumor slope (HU/s)	2.0 ± 0.6	2.9 ± 1.1	*p* < 0.001 *
Tumor peak enhancement (HU)	46.8 ± 12.2	69.9 ± 22.1	*p* < 0.001 *
Enhancement difference (ΔHU)	35.3 ± 19.4	12.0 ± 9.1	*p* < 0.001 *

* Tested using the Mann–Whitney U test.

## Data Availability

The data presented in this study are available upon request from the corresponding author, depending on ethics board approval. The data are not publicly available because of data protection legislation.
